# Improved viability and fertility of frozen-thawed dog sperm using adipose-derived mesenchymal stem cells

**DOI:** 10.1038/s41598-020-61803-8

**Published:** 2020-04-27

**Authors:** Ahmad Yar Qamar, Xun Fang, Min Jung Kim, JongKi Cho

**Affiliations:** 1 Laboratory of Theriogenology, College of Veterinary Medicine, Chungnam National University, Daejeon, 34134 Republic of Korea; 2 Department of Clinical Sciences, College of Veterinary and Animal Sciences, Jhang 35200, Sub-campus University of Veterinary and Animal Sciences, Lahore, 54000 Pakistan; 3 Department of Theriogenology and Biotechnology, College of Veterinary Medicine, Seoul National University, 1 Gwanak-ro, Gwanak-gu, Seoul 08826 Republic of Korea

**Keywords:** Spermatogenesis, Spermatogenesis, Stem-cell research, Stem-cell research

## Abstract

Cryopreservation procedures negatively affect the quality traits of sperm, causing certain changes at structural and molecular levels due to thermal, mechanical, osmotic, and oxidative damage. The objective of this study was to examine the potential of canine adipose**-**derived mesenchymal stem cells (Ad**-**MSCs) for providing protection to the dog sperm against cryo-damage. Canine Ad**-**MSCs were selected on the basis of the significantly higher gene expression for different proteins actively involved in the cell repair including annexin 1 (*ANX1*), histone H3 (*H3*) and high mobility group B (*HMGB*) protein compared to skin fibroblasts. Semen was collected from four healthy dogs by digital manipulation. The washed pooled ejaculates were diluted with buffer 2 (extender) supplemented without Ad**-**MSCs (Control), with 2.5 × 10^6^ Ad**-**MSCs/mL (Group 1) or with 5 × 10^6^ Ad**-**MSCs/mL (Group 2). Group 1 exhibited significantly higher post-thaw motility, live sperm, intact plasma membrane and normal acrosomes than the other groups. Additionally, Group 1 showed significantly higher expression levels of genes related to the repair of membranes (*ANX1*, dysferlin; *DYSF*, and fibronectin; *FN1*) and chromatin material (*H3* and *HMGB*). Protein expression of ANX1, H 3, and FN1 was also statistically more in Group 1 than in Control. The results confirm that canine Ad**-**MSCs can effectively preserve the quality of frozen-thawed sperm by a reduction in cryoinjury. At an appropriate concentration, Ad**-**MSCs significantly improve the quality of post-thaw dog sperm.

## Introduction

Semen cryopreservation is regarded as the most important step of artificial insemination which is the most widely adopted assisted reproductive technology (ART) in canine practice^1^. It can facilitate the storage of the genetic material for an extended period, conserve the elite individual’s fertility and serve as a useful tool for preserving the endangered species. In addition, it has greatly benefited the animal**-**based industry by reducing the cost and the stress associated with transportation, overcoming the quarantine restrictions and breeding issues (e.g., aggressive behavior and size issues)^2^.

Despite many attempts to improve freezing agents and techniques, reduction in semen quality still remains the major issue associated with freezing procedures. During freezing the quality-related traits of sperm are highly compromised^3^. Sperm undergoes certain detrimental changes at the structural and molecular levels as a result of thermal, mechanical, osmotic, and oxidative damage^4,5^. The sperm that survives during freezing procedure suffers a reduction in fertility^6^ and this has been linked with damage that adversely affects viability, motility, plasma membrane, acrosome and chromatin material^7^. In addition, the activation of apoptotic pathways results in the fragmentation of sperm cell DNA^8^. These changes ultimately contribute to an overall reduction in the fertility of sperm.

Current protective modifications employed to minimize the above mentioned damaging effects involve the use of different types of extenders^9^, variations in the freezing protocols^10^, and supplementation of extenders with nutrients or antioxidants^11–16^. However, the rates of whelping using cryopreserved semen (50.0–70.8%) are still lower in comparison with the fresh semen (81.8–83.7%)^17^. The reasons behind the lower fertility of frozen semen may be the primary or secondary damaged sperm that requires immediate regeneration and recovery. In addition, the chemical additives used for supplementation have been also associated with the issues of cytotoxicity^8,18^. Therefore, the repair of injured sperm could be a determining factor for improving the fertility of canine using frozen semen.

Mesenchymal stem cells (MSCs) are believed to be an integral part of regenerative medicine. Recently, research has highlighted their potential role in repair mechanisms occurring at cellular level^19^. The repair of the damaged tissue is believed to be governed by the multipotent nature of MSCs, and the associated paracrine mechanisms involving the secretion of different signaling factors, mainly proteins^19,20^. These proteins modulate the immune response and facilitate regeneration mechanisms by promoting mitosis and angiogenesis but at the same time suppressing apoptosis and scarring^19^. These include basic fibroblast growth factor (bFGF), keratinocyte growth factor (KGF), stromal**-**derived factor-1 (SDF-1), monocyte chemotactic protein**-**1 (MCP**-**1), insulin**-**like growth factor**-**1 (IGF**-**1), transforming growth factor**-**β (TGF**-**β), platelet**-**derived growth factor (PDGF), interleukin**-**8 (IL**-**8) and vascular endothelial growth factor (VEGF)^21,22^. In addition, temperature reduction increases the differentiation potential of MSCs along with a reduction in apoptosis and oxidative stress^23^. The reduced oxidative stress was due to the decrease in the reactive oxygen species, nitric oxide, thiobarbituric acid reactive substances, carbonyl, and lipofuscin production levels^23^.

The biosynthetic capability of sperm is limited^24,25^ serving as the main obstacle in the way of self-repair. Many external factors control sperm function by acting through the surface and membrane components^26^. Therefore, we hypothesized that the use of MSCs in semen cryopreservation may be an effective biological approach to enhance the fertility and viability of sperm by supporting repair mechanisms through the secretion of different proteins. To the best of our knowledge, this is the first study aimed to find out whether canine adipose**-**derived MSCs (Ad**-**MSCs) can improve post-thaw fertility and survival of dog sperm. In addition, identification of the factors involved in the repair mechanisms was also attempted.

## Results

### Comparison of gene expression between canine Ad-MSCs and skin fibroblasts

**Figure 1:**
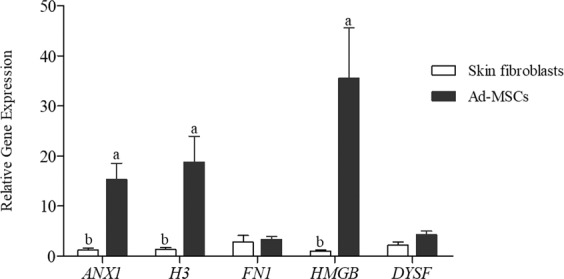
Gene expression of annexin 1 (*ANX1*), histone H3 (*H3*), fibronectin (*FN1*), high mobility group protein B (*HMGB*) and dysferlin (*DYSF*) by RT-qPCR in canine Ad**-**MSCs compared with skin fibroblasts (mean ± SEM). Different lowercase letters a, or b, indicate a significant difference at *P* < 0.05.

The expression levels of different genes related to the repair of the membrane (*ANX1*, *FN1*, and *DYSF*) and chromatin material (*H3* and *HMGB*) among canine Ad**-**MSCs and skin fibroblasts is shown in Fig. 1. The expression levels of *ANX1*, *H3*, and *HMGB* genes were significantly higher in Ad**-**MSCs than skin fibroblasts. However, the expression level of *FN1* and *DYSF* was not statistically different among the Ad**-**MSCs and skin fibroblasts.

### Effect of Ad-MSCs on motility and kinetic parameters

Post**-**thaw motility varied significantly among the groups (Table 1). The post-thaw motility in Group 1 (55.7 ± 1.0%) appeared to be significantly higher than in Group 2 and Control (48.8 ± 1.8% and 43.2 ± 1.0%, respectively). Sperm linearity did not show any statistical variation among the groups, but Group 1 (26.9 ± 0.6%) showed an increasing trend than Group 2 and Control (23.3 ± 0.9% and 23.9 ± 1.8%, respectively). Sperm straightness was not statistically different between the Group 1 and 2 (49.6 ± 1.6% and 49.3 ± 0.6%; respectively) but was significantly higher compared to the Control (45.1 ± 0.5%). The amplitude of lateral head displacement (ALH) was significantly higher in Group 1 (3.4 ± 0.1%) as compared to Control (2.8 ± 0.1%), whereas Group 2 (2.9 ± 0.1%) showed no statistical difference from either of Groups 1 and Control. The post-thaw motility was strongly but positively correlated (P < 0.01) with ALH, membrane integrity, live sperm percentage, normal acrosome integrity, and chromatin integrity (Table 2).

**Table 1:** Effects of addition of canine Ad-MSCs in different concentrations (control; without Ad-MSCs, Group 1; 2.5 × 10^6^ cells/mL, Group 2; 5 × 10^6^ cells/mL) on post-thaw semen quality of beagle dogs.

Group	Motility (%)	Linearity (%)	Straightness (%)	ALH (µm)	Live sperm (%)	Membrane Integrity (%)	Normal acrosome (%)	Normal Chromatin (%)
Control	43.2 ± 1.0^c^	23.9 ± 1.8	45.1 ± 0.5^b^	2.8 ± 0.1^b^	43.7 ± 1.1^b^	45.4 ± 0.8^c^	44.3 ± 0.7^b^	65.3 ± 0.9^b^
Group 1	55.7 ± 1.0^a^	26.9 ± 0.6	49.6 ± 1.6^a^	3.4 ± 0.1^a^	52.8 ± 0.8^a^	54.6 ± 0.4^a^	52.9 ± 0.5^a^	72.9 ± 0.7^a^
Group 2	48.8 ± 1.8^b^	23.3 ± 0.9	49.3 ± 0.6^a^	2.9 ± 0.1^ab^	41.9 ± 1.6^b^	48.6 ± 0.9^b^	50.1 ± 1.9^a^	68.9 ± 1.1^b^

ALH, amplitude of lateral head displacement. Values with different superscript letters in a column differ significantly (P < 0.05).

**Table 2:** Correlation coefficient between different quality-related parameters of post-thaw semen.

No.	Variables	Correlation between values							
		1	2	3	4	5	6	7	8
1	Post-thaw motility (%)	—	0.28	0.50	0.94**	0.88**	0.74**	0.78**	0.78**
2	Linearity (%)	—	—	−0.00	0.289	0.48	0.54	0.21	0.28
3	Straightness (%)	—	—	—	0.27	0.55	0.31	0.67*	0.67*
4	ALH (µm)	—	—	—	—	0.76**	0.74**	0.62*	0.60*
5	Membrane Integrity (%)	—	—	—	—	—	0.73**	0.89**	0.88**
6	Live sperm (%)	—	—	—	—	—	—	0.41	0.65*
7	Normal Acrosome (%)	—	—	—	—	—	—	—	0.82**
8	Normal Chromatin (%)	—	—	—	—	—	—	—	—

**Correlation coefficient is significant at the 0.01 level (2-tailed).

*Correlation coefficient is significant at the 0.05 level (2-tailed).

### Effect of Ad-MSCs on viability and membrane integrity

The post**-**thaw live sperm percentage was significantly more in Group 1 (52.8 ± 0.8%) as compared to Group 2 and Control (41.9 ± 1.6% and 43.7 ± 1.1%, respectively; Table 1). However, the latter two groups did not vary statistically. The HOS test results showed the percentage of sperm with an intact plasma membrane differed significantly between the groups, with Group 1 (54.6 ± 0.4%) being statistically superior in comparison with Group 2 (48.6 ± 0.9%) and Control (45.4 ± 0.8%) (Table 1). Live sperm percentage and membrane integrity were strongly and positively correlated (P < 0.01) with each other. In addition, membrane integrity was strongly and positively correlated (P < 0.01) with normal acrosome integrity, and chromatin integrity (Table 2). However, live sperm percentage was positively correlated (P < 0.05) with chromatin integrity (Table 2).

### Effect of Ad-MSCs on acrosomal integrity

The results of the post-thaw semen analysis of frozen semen shown in Table 1 indicated that the percentages of sperm with normal acrosomal region differed statistically among the different groups. The post-thaw semen analysis showed significantly higher sperm count with intact acrosome in Group 1 and Group 2 (52.9 ± 0.5% and 50.1 ± 1.9) than Control (44.3 ± 0.7%) (Table 1). The acrosomal integrity was strongly but positively correlated (P <  0.01) with chromatin integrity (Table 2).

### Effect of Ad-MSCs on chromatin integrity

Results of the acridine orange test showed that a significantly higher percentage (72.9 ± 0.7%) of frozen**-**thawed sperm in Group 1 had normal chromatin compared with Group 2 (68.9 ± 1.1%) and Control (65.4 ± 0.9%) (Table 1).

### Effect of Ad-MSCs on gene expression

**Figure 2:**
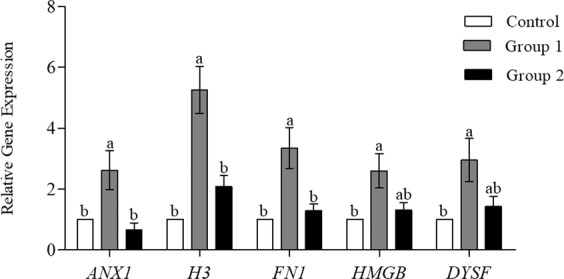
Gene expression of plasma membrane repair related genes annexin 1 (*ANX1*), fibronectin (*FN1*), dysferlin (*DYSF*) and chromatin material repair related genes high mobility group protein B (*HMGB*), histone H3 (*H3*) by real time RT**-**qPCR in Control (without Ad**-**MSCs), Group 1 (2.5 × 10^6^ cells/mL of buffer 2) and Group 2 (5 × 10^6^ cells/mL of buffer 2) of cryopreserved sperms (mean ± SEM). Different lower case letters a, or b indicate a significant difference at *P* < 0.05.

Results indicated a significant enhancement of the expression levels of different genes related to the repair of the membrane (*ANX1*, and *FN1*) and chromatin material (*H3*) in Group 1 sperm as compared with the other groups (Fig. 2). However, no significant difference was witnessed between Group 2 and Control. The expression levels of *DYSF* and *HMGB* genes showed a different pattern of expression, Group 2 being statistically similar to both Groups 1 and Control, but the expression in Group 1 was significantly higher than Control.

### Western blot analysis

**Figure 3:**
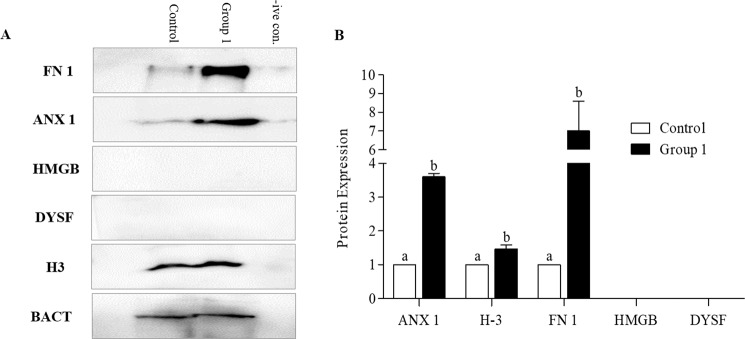
Expression of proteins analysed by western blot with corresponding antibodies. (**A**) Representative western blot images of annexin 1 (ANX1), histone H3 (H3), fibronectin (FN1), high mobility group protein B (HMGB) and dysferlin (DYSF) proteins and (**B**) quantification of ANX1, H3, FN1, HMGB and DYSF protein expression levels in the Control and Ad**-**MSCs treated cryopreserved canine semen (Group 1). Data obtained by densitometry showing optical densities relative to the expression in Control. Beta actin expression level served as the loading control. Data are expressed as the mean ± SEM. Different lower case letters a, or b, indicate a significant difference at *P* < 0.05.

Based on the results obtained for different parameters of semen analysis and expression levels of different genes, protein expression was evaluated among Group 1 with optimal concentration of Ad**-**MSCs and Control using western blot: proteins related to the repair of membrane (ANX1, FN1, and DYSF) and chromatin material (histone H3, and HMGB). Beta**-**actin served as an Internal Control. Western blot images of protein expression levels for Group 1 and Control are presented in Fig. 3A and Supplementary Fig. S1. Protein expression was semi**-**quantitatively evaluated using densitometry (Fig. 3B). Results elucidate that the expression levels of histone H3, FN1 and ANX1 were significantly higher in Group 1 than Control. However, the HMGB and DYSF failed to express in either of the group.

## Discussion

Studies have demonstrated the potential role of MSCs in various biological processes like contributing to cell generation, survival, and alteration of cellular phenotype^27–29^. MSCs govern the repair mechanisms occurring at cellular and molecular levels by communication through the release of different factors mostly proteins^30^. In this study, we investigated the potential role of MSCs in the repair of the plasma membrane and chromatin material of dog sperm following cryopreservation. Canine Ad**-**MSCs were selected due to their abundant availability, and significantly better gene expression levels as compared to canine skin fibroblasts (Fig. 1). Canine Ad**-**MSCs show higher expression levels of *ANX1*, *H3*, and *HMGB* due to their un**-**differentiated nature, multi**-**lineage differentiation both *in vivo* & *in vitro*^20^, and better proliferation rate as compared to fibroblasts.

This study elucidated the capacity of canine Ad**-**MSCs to enhance the post**-**thaw survival rate of dog sperm through ameliorated repair mechanisms, occurring at cellular as well as molecular levels initiated in response to cryoinjury. It is well**-**known that the variations of temperature during cryopreservation have an inverse effect on function (motility) and structure (plasma membrane, mitochondria, and nucleus) of sperm resulting in impaired fertility^31–33^. Significant improvements in the post**-**thaw sperm motility and viability parameters of Group 1, (2.5 × 10^6^ Ad**-**MSCs per mL of buffer 2). However, the higher concentration of Ad**-**MSCs in Group 2 resulted in negative impacts on quality**-**related parameters (Table 1). The sperm in Group 2 suffered serious damage due to increased packing proportion during cryopreservation. As cells become more closely packed, new factors are produced and further protection is required for sustaining cellular viability^34–37^. Similarly, the post**-**thaw survival rate of red blood cells^34,38^, and hepatocytes^39^, has been negatively influenced by higher concentrations during cryopreservation.

The plasma membrane is the outermost structure of the cell acting as a barrier against foreign agents to maintain the cellular structures and functions. Sperm’s plasma and acrosomal membranes are critically essential for survival following thawing and are termed as the primary sites of change following cryopreservation^26^. The structural integrity of the plasma membrane is highly correlated with motility and viability of sperm^40^. Similarly in our study, the Group 1 sperm that showed enhanced integrity of plasma and acrosomal membrane also exhibited significantly higher motility and kinetic parameters (Table 1). The possible reason for improved integrity might be the proteins secreted by Ad**-**MSCs such as annexin, dysferlin, and fibronectin that are generally involved in repair mechanisms at the cellular level. Annexins have a role in exocytosis, endocytosis, aggregation, membrane fusion^41^, and Ca^+2^**-**dependent membrane binding makes them well suited for repair of membranes^42^. Annexin 1 has been reported to protect rat retinal ganglion cells against serum-derived apoptosis^43^. In addition, annexin 1 and 2, along with dysferlin protein have been proposed to be involved in the Ca^+2^**-**dependent sarcolemmal repair of skeletal myocytes^44,45^. Fibronectin regulates cellular processes and the maintenance and repair of damaged tissue^46^. In addition, it is a seminal plasma protein with a variety of reproductive functions including activation of proteasomes, acrosomal reaction^47^, capacitation^48^, gamete interaction^49^, and embryonic development^50^. Our assumption that improved membrane integrity was based on high gene expression levels of proteins was well supported by the significantly higher levels of annexin, dysferlin and fibronectin seen in the Group 1 sperm (Fig. 2). We also compared protein expression levels in Group 1 and Control through western analysis, confirming statically higher expression level of annexin and fibronectin proteins in Group 1 (Fig. 3A).

Chromatin integrity appears to be imperative for the proper functioning of sperm^51^. Excessive damage to sperm DNA compromises fertility^52–55^ and serves as a predisposing factor for various genetic disorders, birth defects, and early**-**life cancers in offspring^53,56^. Histone H3 is one of the core histone proteins, wrapping the DNA of mammalian sperm and its variants have been shown to mediate function like DNA repair^57^. Similarly, HMGB participates actively in multiple types of nuclear^58^ and mitochondrial DNA repair^59^. In our study, Group 1 sperm exhibited a significantly reduced fragmentation of sperm DNA as compared to other groups (Table 1). Quantitative analysis of gene expression among the different groups showed statistically higher expression levels of *H3* and *HMGB* in Group 1 sperm (Fig. 2). The significantly higher expression level of histone H3 protein in Group 1, supported our assumption (Fig. 3B).

Results exhibited that the canine Ad**-**MSCs have the potential to enhance the quality of cryopreserved dog sperm. Optimal levels of Ad**-**MSCs positively influenced all the quality**-**related traits including motility, viability, membrane integrity, acrosomes and chromatin in post**-**thaw sperm. Canine Ad**-**MSCs supported repair mechanisms by communicating with the damaged sperm *via* production of different proteins that serve as an integral part of repair machinery. Further studies would be desirable to determine other factors secreted by Ad**-**MSCs that may play an active role in preserving sperm quality, and *in vivo* trials would also help to clarify the usefulness of Ad**-**MSCs in semen cryopreservation. In addition, the use of Ad-MSCs supplemented semen for artificial insemination may also be helpful in resolving the issues associated with the female reproductive tract due to their regenerative and multipotent nature.

## Methods

In this experiment, all chemicals utilized were acquired from Sigma-Aldrich (St. Louis, MO, USA) unless otherwise mentioned.

### Animals used

Four healthy mature male beagles, 2**–**3 years of age, and 7**–**11 kg weight were utilized for semen collection. A separate indoor cage was used for housing each individual subject, furnished with all the facilities related to animal care and procedures standardized by the Committee for Accreditation of Laboratory Animals at Seoul National University. All procedures were performed according to the guidelines specified in “Guide for the Care and Use of Laboratory Animals at Seoul National University (approval no. SNU**-**180731**–**2)”.

### Experimental design

In experiment 1, the selection of suitable cell type was performed on the basis of comparison of the relative expression levels of different genes among canine Ad**-**MSCs and skin fibroblasts through RT**-**qPCR. In experiment 2, the optimal concentration of canine Ad**-**MSCs required to preserve the motility and viability of frozen**-**thawed semen was determined. In experiment 3, the mechanisms responsible for repairing the plasma membrane and chromatin material of the sperm were determined. For this purpose, the expression levels of different genes and proteins in post**-**thaw sperm were compared among groups without Ad**-**MSCs (Control) and with an optimal concentration of Ad**-**MSCs.

### Culture of canine Ad-MSCs

**Figure 4:**
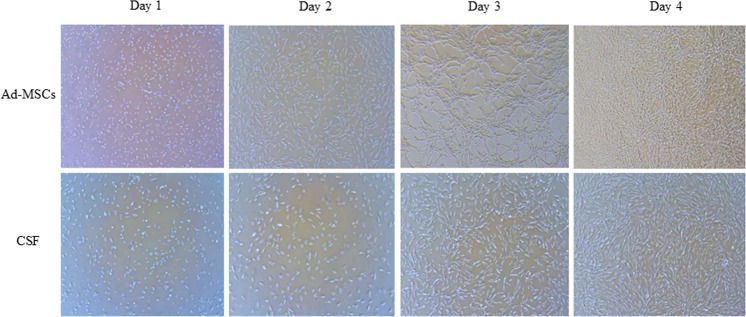
Morphology of canine adipose derived mesenchymal stem cells (Ad**-**MSCs) and canine skin fibroblasts (CSF) after every 24 h of culture up to 80–90% confluence (Magnification: 50×).

Characterized canine Ad**-**MSCs^60^ and AMSC media (the canine Ad**-**MSCs culture medium) were obtained from Naturecell Co., Ltd (Seoul, Republic of Korea). Ad-MSCs were cultured following the same procedures as previously explained^61^. Briefly, frozen Ad**-**MSCs were thawed in a water bath at 37 °C, washed and re**-**suspended in AMSC media. Following washing, the cells were cultured in a cell culture dish and incubated in a humidified environment of 5% CO_2_ at 37 °C until 80**–**90% confluence (Fig. 4). At passage 3, the Ad**-**MSCs fraction was recovered using 0.05% trypsin**-**EDTA (Gibco by Life Technologies, Canada), and used in the cryoprocessing of semen.

### Semen collection and preparation

Sperm rich**-**fractions were collected twice a week. The selection of semen ejaculates for the experiments was based on the same criteria as explained previously^61^. Briefly, the ejaculates with a sperm count of ≥100 × 10^6^/mL, motile sperm ≥70%, and ≥80% of normal morphology possessing viable sperm were pooled together. Sperm were separated from seminal fluids by the spinning of the pooled ejaculate at 100× g for 1 min at room temperature and recovered the supernatant. The same volume of buffer 1 [24 g/L tris (hydroxymethyl) aminomethane, 14 g/L citric acid, 8 g/L of fructose, and 0.15 g/L kanamycin sulfate in distilled water (pH 6.6, 290 mOsmol)] was added to the recovered supernatant and was subjected to centrifugation at 700× g speed for 5 min. Resuspended sperm pellet using buffer 1 to achieve a concentration of 200 × 10^6^ sperm/mL. The final concentration of 100 × 10^6^ sperm/mL was attained by adding a suitable volume of buffer 2 (6% (v/v) glycerol, 40% (v/v) egg yolk, and 54% (v/v) buffer 1)^62^, supplemented without Ad**-**MSCs (Control), with 2.5 × 10^6^ Ad**-**MSCs/mL (Group 1) or with 5 × 10^6^ Ad**-**MSCs/mL (Group 2). The mixing of buffer 2 was carried out following a multi-step loading protocol, mixing final sperm fraction with 14%, 19%, 27% and 40% of buffer 2 (total calculated volume) at an interval of 30 sec. The Control and Groups 1 & 2 were prepared and incubated for 45**–**60 min in a CO_2_ incubator at 37 °C prior to sperm collection.

### Freezing and thawing of sperm

Following extension, the semen was filled into 0.25 mL straws (Minitub, Germany). Sealed straws were kept at 4 °C for 1 h to carry out the equilibration procedure, followed by freezing of semen by horizontally placing the straws 2 cm above liquid nitrogen (LN_2_) for 15–20 min and finally stored at −192 °C using LN_2_. Post-thaw evaluations were performed a week after cryopreservation of semen. Thawing of semen was performed in a water bath at 37 °C for 30 sec, then diluted with buffer 1 (1:5, semen: buffer 1) stepwise by 14%, 19%, 27% and finally 40% of the total volume.

### Separation of sperm from Ad-MSCs

Post**-**thaw sperm were separated from the Ad**-**MSCs using a percoll density gradient procedure, as previously explained^63^. Briefly, 1 mL of post**-**thaw semen from each group was pipetted gently onto a double-layered column of 45%/90% Percoll (Pharmacia) in a conical tube of 15 mL capacity. The tubes were then centrifuged at 400 × g for 20 min and the fluid was recovered from phase C, D, and E for further analysis of different parameters. These phases have been reported to possess higher percentages of sperm output, motility and fewer number of Ad**-**MSCs^63^.

### Assessment of motility and kinetic parameters

Post-thaw motility and kinetic parameters were examined following the same procedure as previously explained^61^. Briefly, a semen drop (10 µL) was placed on a pre**-**warmed glass slide and mounted with a cover glass. Sperm were screened in 5 different fields and 200 motile sperm were tracked for the assessment of the kinetic parameters employing a sperm analysis imaging system (FSA2011 premium edition version 2011; Medical Supply, Korea).

### Assessment of viability of sperm

Eosin**-**nigrosin staining procedure was used to examine the percentage of viable sperm as previously explained^64,65^. Briefly, a semen drop (5**–**10 µL) was mixed with an equal amount of the stain on a pre**-**warmed glass slide. A thin smear was made on a new slide using the semen**-**stain mixture and dried in air. Examined 200 hundred sperm per slide and classified as possessing an intact membrane (white staining) or a non**-**intact membrane (pink staining).

### Assessment of the plasma membrane integrity

The integrity of the sperm plasma membrane was accessed by hypo**-**osmotic swelling (HOS) assay^65^, using a HOS solution of 190 mOsmol/kg. Fifty µL of post-thaw semen from each group was mixed with 500 µL of HOS solution and incubated at 37 °C for 30 min. Following incubation, a 5-µL drop of the mixture was placed on a clean, warm glass slide and 200 sperm were examined for their swelling ability using a phase**-**contrast microscope (Eclipse Ts 2, Nikon). The sperm with intact plasma membrane showed swelling, indicated by the coiled tail.

### Assessment of acrosomal membrane integrity

The intactness of sperm acrosome was analyzed using the fluorescein isothiocyanate-conjugated peanut agglutinin (FITC-PNA) staining method. Semen drop (30 µL) was used to prepare smears on glass slides. Smears were air-dried and fixed by dipping the smears in absolute methanol at 20**–**22 °C for 10 min. After drying the smears, staining was performed by spreading 30 µL of FITC**-**PNA solution (100 µg/mL) in phosphate buffer saline (PBS). Subsequently, slides were incubated in a dark moist chamber at 37 °C for 30 min. Finally, smears were rinsed using PBS, air-dried and immediately observed using an epifluorescence phase**-**contrast microscope (Eclipse Ts 2, Nikon). The acrosome-intact sperm were observed with strong green fluorescence and the percentage of fluorescent acrosome**-**intact sperm was counted in at-least 200 sperms per slide.

### Assessment of sperm chromatin quality

**Figure 5:**
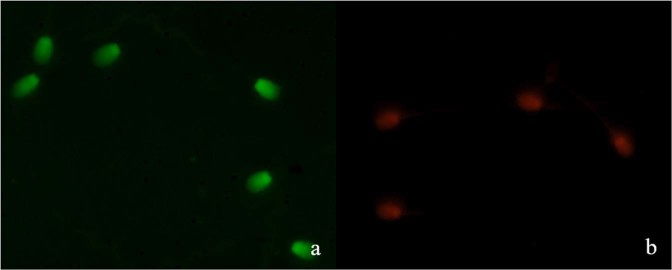
Image of acridine orange**-**stained sperm showing normal (**a**) and denatured (**b**) sperms.

Washed separated sperm were smeared onto a clean glass slide, dried in the air, followed by overnight fixation using Carnoy’s solution (3:1, methanol: glacial acetic acid) and dried again in the air. The slides were dipped into a solution of 0.1 M citric acid solution (pH 2.5) at room temperature for 5 min and then rinsed repeatedly with distilled water. The smeared slides were then stained for 5 min in acridine orange solution (0.2 mg/mL water) and washed with distilled water. Analysis of the slides was carried out fluorescence microscopy at 1,000× magnification of Zeiss microscope and nuclei from 200 sperm were examined. Sperm with normal chromatin material showed green**-**fluorescence at the head (bicatenary DNA, Fig. 5a), whereas abnormal chromatin material showed red**-**fluorescence at the head (monocatenary, DNA, Fig. 5b).

### Assessment of gene expression

Quantitative polymerase chain reaction (qPCR) was conducted both for comparison among cells (Ad**-**MSCs vs. skin fibroblasts) and different semen groups (Control, Group 1, and Group 2) as previously described^1^. Briefly, RNA was extracted from cells and post-thawed sperm obtained from five pairs of straws from each treatment group and Control. For the assessment of transcript abundance, oligonucleotide primer sequences, (listed in Table 3) were used, employing real-time qPCR (RT-qPCR). Extraction of RNA was conducted using Trizol reagent (Invitrogen, USA), followed by complementary DNA synthesis using Maxime RT PreMix (Intronbio, Korea) following the manufacturer’s protocol. RT**-**qPCR was utilized to examine the expression of plasma membrane repair**-**related genes (annexin 1, *ANX1*; dysferlin, *DYSF*; and fibronectin 1, *FN1*) and the chromatin material repair**-**related genes (high mobility group protein B, *HMGB*; and histone H3, *H3*;) using Step One Plus Real-Time PCR System (Applied Biosystems, USA) and the quantification of expression level of each target gene relative to that of the internal gene beta-actin (*BACT*) was conducted employing the equation, R = 2^−[ΔCt sample − ΔCt control]^.

**Table 3:** Primer sequences used for gene expression analysis.

Gene	Primer sequence (5′-3′)	Product size (bp)	NCBI accession no.
*BACT*	F: GAGGCATCCTGACTCTGA	87	XM_544346.3
	R: TCTGGCACCACACTTTCT		
*ANX1*	F: GAAGCTCTGAAGAAAGCCC	128	NM_001286970.1
	R: GTGTCTTCATCAGTTCCAAGG		
*H3*	F: CGGTGACTGACACGCGAC	136	XM_022404950.1
	R: GTTGGAGCAGGCCTTGAACC		
*FN1*	F: ATAGCTGGCTGTTACGAC	74	XM_022415242.1
	R: GCATTTCCCAGGTAGGTG		
*HMGB*	F: ATATTGCTGCGTACCGAG	64	XM_022409535.1
	R: TCAGCCTTGACAACTCCC		
*DYSF*	F: TGGATCAGAGTGGCGTCC	127	XM_003432223.4
	R: GACAGCAGCTTTCTGGCT		

F, forward; R, reverse. Beta actin (*BACT*), annexin 1 (*ANX1*), histone H3 (*H3*), fibronectin 1(*FN 1*), high mobility group protein B (*HMGB*), and dysferlin (*DYSF*).

### Western blot analysis

Western analysis of post**-**thaw semen was conducted using individual samples from treatment Group 1 and Control (n = 6 per group). Protein extraction from the semen samples was carried out using Protein Extraction Solution (PRO-PREP^TM^, iNtRON biotechnology, Korea). In each group, a total of 25 µg protein was added in 2X Laemmli sample buffer (Bio**-**Rad laboratories, USA) in the ratio 1:2. Proper mixing was achieved through gentle pipetting, followed by the boiling of samples for 10 min at 100 °C. Loaded samples into a 10% (w/v) polyacrylamide gel and electrophoresis was carried out at a constant voltage (90 V) for 2 h. Precision Plus Protein^TM^ Standards Dual Color (Bio-Rad) was utilized as a molecular weight marker. The separated proteins were transferred (15 V for 80 min) to methanol**-**activated polyvinylidene difluoride (PVDF) membranes (Immobilon-P transfer membranes, Merck KGaA, Darmstadt, Germany) and blocked using 5% (w/v) nonfat dry milk (Bio-Rad) solution at room temperature for 90 min. Membranes were exposed at 4 °C to primary goat polyclonal anti**-**annexin A1 (Abcam, UK), rabbit polyclonal anti-acetyl**-**histone H3 (Millipore, USA), rabbit polyclonal anti**-**fibronectin (Abcam, UK), mouse monoclonal anti-HMG**-**1 (Sigma, USA) rabbit polyclonal anti**-**dysferlin, and mouse monoclonal anti**-**beta actin (Abcam, UK) followed by incubation in respective horseradish peroxidase-conjugated anti-goat, rabbit or mouse (Abcam, UK), at the same temperature for 2 h. Membranes were incubated with Clarity^TM^ Western ECL substrate (Bio-Rad) for 5 min, and the chemiluminescence signals were read using Chemiluminescence imaging**-**Fusion SOLO software (Vilber Lourmat, France). The quantification of band densities was carried out employing Image J software (Version 1.41; National Institutes of Health, Bethesda, MD, USA).

### Statistics

All values were presented as mean ± SEM, and a *P-*value of <0.05 was taken as an indication of statistical significance. An independent sample *t***-**test was used for comparing the gene expression levels among canine Ad**-**MSCs and skin fibroblasts. One**-**way analysis of variance (ANOVA) and Tukey’s Multiple Comparison Test were utilized for comparing the treatments and Control group. The correlation between different parameters was also determined^66^. One sample *t***-**test was utilized for comparing the results of western blot analysis from the Control and optimal Ad**-**MSCs concentration group. Analysis of data was conducted using SPSS 21.0 software (SPSS Inc., Chicago, USA).

### Ethics approval and consent to participate

This study was approved by the Ethics Committee on Animal Use (approval no. SNU-180731-2-1) of the Seoul National University (Seoul, Republic of Korea).

## Supplementary information

Supplementary information
