# Supplementary material for: Improved viability and fertility of frozen-thawed dog sperm using adipose-derived mesenchymal stem cells
Source: Sci Rep. 2020 Apr 27;10:7034. doi: 10.1038/s41598-020-61803-8 (PMC7184611; doi:10.1038/s41598-020-61803-8)
Supplement: Supplementary file 1 — Supplementary information [file 41598_2020_61803_MOESM1_ESM.pdf]

Supplementary Material

Improved viability and fertility of frozen-thawed dog sperm using adipose-derived mesenchymal stem cells

Ahmad Yar Qamar[1], Xun Fang[1], Min Jung Kim[2, *], and Jongki Cho[1,*]

* Corresponding author: cjki@cnu.ac.kr

[2, *] Co-corresponding author: tinia19@snu.ac.kr

1. Supplemental files

Supplementary Figure Legends

Supplementary Figure S1. Effect of canine adipose-derived mesenchymal stem cells (Ad-MSCs) on cryopreservation of dog semen. Representative western blot images of fibronectin (FN1), annexin 1 (ANX1), high mobility group protein B (HMGB), dysferlin (DYSF) proteins, histone H3 (H3), and Beta-actin (BACT) protein expressed in post-thaw canine sperm. Where, Control; 1, Group 1; 2.5×10^6 Ad-MSCs/mL, negative control; (-).

Supplementary Figure S1

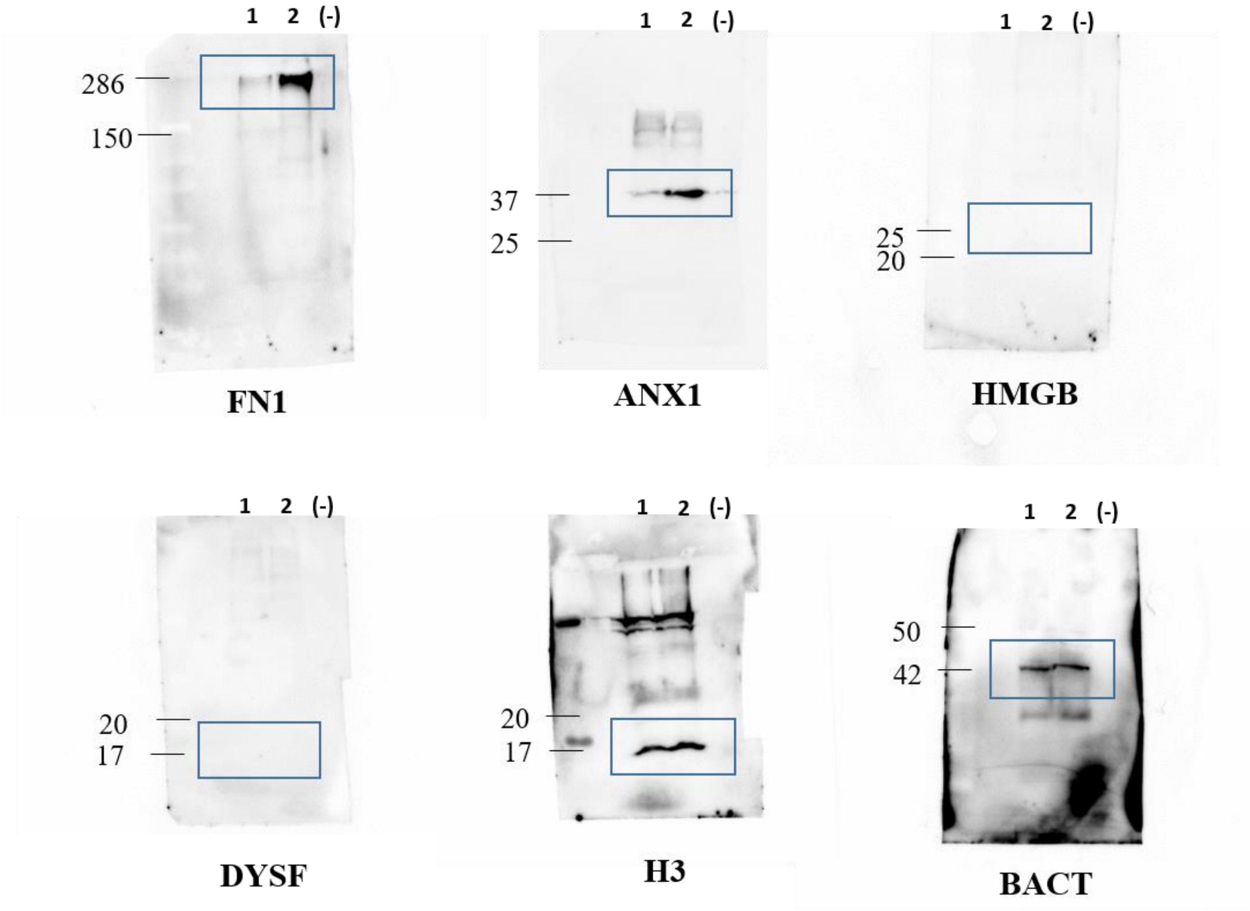

Supplementary Figure S1. Effect of canine adipose-derived mesenchymal stem cells (Ad-MSCs) on cryopreservation of dog semen. Representative western blot images of fibronectin (FN1), annexin 1 (ANX1), high mobility group protein B (HMGB), dysferlin (DYSF) proteins, histone H3 (H3), and Beta-actin (BACT) protein expressed in post-thaw canine sperm. Where, Control; 1, Group 1; 2.5 × 10^6 Ad-MSCs/mL, negative control; (-).
